# A new mutation-independent approach to cancer therapy: Inhibiting oncogenic RAS and MYC, by targeting mitochondrial biogenesis

**DOI:** 10.18632/aging.101304

**Published:** 2017-10-27

**Authors:** Bela Ozsvari, Federica Sotgia, Michael P. Lisanti

**Affiliations:** ^1^ Translational Medicine, School of Environment and Life Sciences, Biomedical Research Centre (BRC), University of Salford, Greater Manchester, UK; ^2^ The Paterson Institute, University of Manchester, Withington, UK

**Keywords:** RAS, MYC, cancer stem cells (CSCs), cancer therapy, mitochondrial oxidative stress

## Abstract

Here, we used MCF7 cells as a model system to interrogate how MYC/RAS co-operativity contributes to metabolic flux and stemness in breast cancer cells. We compared the behavior of isogenic MCF7 cell lines transduced with c-Myc or H-Ras (G12V), either individually or in combination. Cancer stem cell (CSC) activity was measured using the mammosphere assay. c-Myc augmented both mammosphere formation and mitochondrial respiration, without any effects on glycolytic flux. In contrast, H-Ras (G12V) synergistically augmented both mammosphere formation and glycolysis, but only in combination with c-Myc, directly demonstrating MYC/RAS co-operativity. As c-Myc is known to exert its effects, in part, by stimulating mitochondrial biogenesis, we next examined the effects of another stimulus known to affect mitochondrial biogenesis, i.e. ROS production. To pharmacologically induce oxidative stress, we used Rotenone (a mitochondrial inhibitor) to target mitochondrial complex I. Treatment with Rotenone showed bi-phasic effects; low-dose Rotenone (1 to 2.5 nM) elevated mammosphere formation, while higher doses (10 to 100 nM) were inhibitory. Importantly, the stimulatory effects of Rotenone on CSC propagation were blocked using a mitochondrial-specific anti-oxidant, namely Mito-tempo. Thus, “mild” mitochondrial oxidative stress, originating at Complex I, was sufficient to pheno-copy the effects of c-Myc, effectively promoting CSC propagation. To validate the idea that mitochondrial biogenesis is required to stimulate CSC propagation, we employed Doxycycline, a well-established inhibitor of mitochondrial protein translation. Treatment with Doxycycline was indeed sufficient to block the stimulatory effects of H-Ras (G12V), c-Myc, and Rotenone on CSC propagation. As such, Doxycycline provides a strong rationale for designing new therapeutics to target mitochondrial biogenesis, suggesting a new “mutation-independent” approach to cancer therapy. In support of this notion, most currently successful anti-cancer agents therapeutically target “cell phenotypes”, such as increased cell proliferation, rather than specific genetic mutations. Remarkably, we demonstrated that Doxycycline inhibits the effects of diverse oncogenic stimuli, of both i) genetic (MYC/RAS) and ii) environmental (Rotenone) origins. Finally, we discuss the advantages of our “Proteomics-to-Genomics (PTG)” approach for *in silico* validation of new biomarkers and novel drug targets. In this context, we developed a new Myc-based Mito-Signature consisting of 3 mitochondrial genes (HSPD1; COX5B; TIMM44) for effectively predicting tumor recurrence (HR=4.69; p=2.4e-08) and distant metastasis (HR=4.94; p=2.8e-07), in ER(+) in breast cancer patients. This gene signature could serve as a new companion diagnostic for the early prediction of treatment failure in patients receiving hormonal therapy.

## INTRODUCTION

Historically, the c-Myc and H-Ras genes have played a major role in our understanding of how genetics contributes to the pathogenesis of human cancers [1]. Originally, both H-Ras and c-Myc were identified as viral oncogenes that were “stolen” from the cellular genome; they were shown to confer the oncogenic potential of RNA tumor viruses [2]. Later, it was directly confirmed that they are indeed derived from normal cellular proto-oncogenes that can be dys-regulated by genetic amplification and/or by specific activating mutations [3].

The concept that multiple genetic changes or “hits” are additive, resulting in a step-wise progression towards cellular transformation, also derives from studies using c-Myc and H-Ras. More specifically, these pioneering studies showed that c-Myc and mutant H-Ras co-operate, in promoting cell transformation and tumori-genesis [4, 5].

However, mechanistically it still remains largely unknown exactly how c-Myc and H-Ras co-operate to promote or facilitate the process of oncogenic trans-formation. Here, we specifically examined their role in the promotion of stem-like characteristics and metabolic phenotypes in cancer cells. For this purpose, we created a panel of MCF7 breast cancer cell lines, transduced with the cDNA's encoding c-Myc or H-Ras (G12V), either individually or in combination. This provides a new epithelial-based isogenic model to study the cellular mechanisms underpinning oncogene co-operation.

Our results indicate that c-Myc and H-Ras (G12V) act synergistically to increase the capacity of cancer cells to undergo anchorage-independent growth and to amplify their energetic activity, effectively increasing both i) mitochondrial respiration (c-Myc) and ii) the glycolytic pathway (H-Ras (G12V)). Importantly, treatment with Doxycycline (a well-established inhibitor of mito-chondrial biogenesis) was sufficient to halt and/or prevent the increases in CSC propagation driven by c-Myc and H-Ras (G12V). Thus, our results suggest that inhibition of mitochondrial biogenesis may be a new “phenotype-based” strategy for “mutation-independent” anti-cancer therapy.

## RESULTS

### MYC-RAS co-operativity increases “stemness”, mitochondrial respiration and glycolytic activity

**Figure 1:**
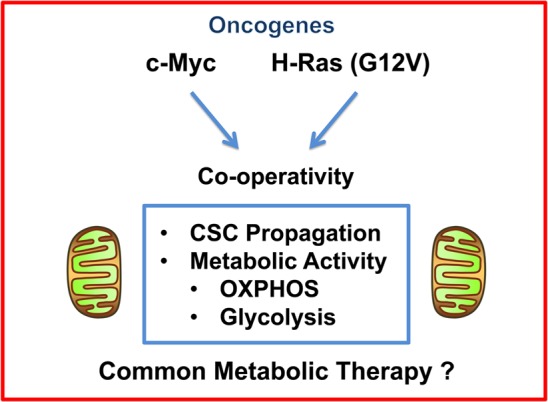
Experimental strategy for understanding MYC/RAS co-operativity in human breast CSCs More specifically, we examined how MYC/RAS co-operation affects cellular metabolism and CSC propagation, allowing for the identification of a common metabolic therapy.

In order to investigate the mechanism(s) underlying MYC-RAS co-operativity, we first created a panel of isogenic MCF7 cell lines using lentiviral vectors (Figure 1). Briefly, MCF7 cells were transduced with either c-Myc alone or H-Ras (G12V) alone, or sequentially transduced with both c-Myc and H-Ras (G12V), using two different selectable markers. A series of empty vector control cell lines were also produced in parallel, to normalize for possible effects due to the process of lentiviral transduction. This battery of cell lines was then subjected to phenotypic characterization.

**Figure 2:**
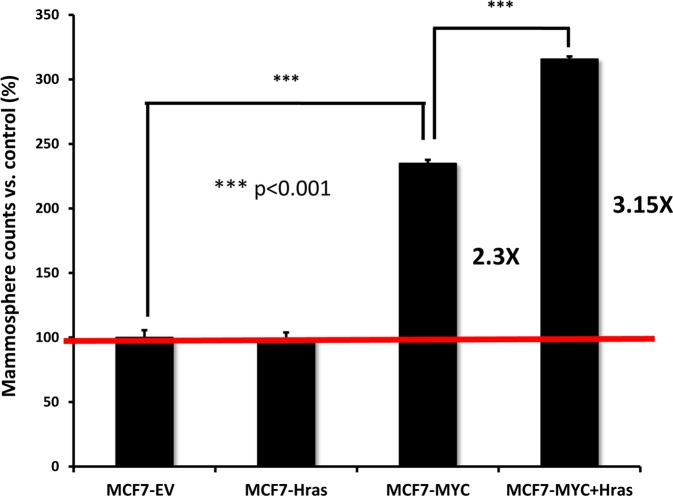
MYC-RAS co-operativity augments mammosphere formation Note that stable transduction with H-Ras (G12V) alone has no effect on mammosphere formation. In contrast, transduction with c-Myc increases mammosphere formation by ∼2.3-fold. Finally, co-transduction with c-Myc and H-Ras (G12V) synergistically increases mammosphere formation by ∼3.15-fold, as compared to vector alone controls. Therefore, MYC-RAS co-operativity augments “stemness” in cancer cells.

Figure 2 shows that oncogenic H-Ras alone surprisingly had no effect on mammosphere formation. Mammo-sphere formation is generally regarded as a measure of CSC activity and/or the ability of cells to undergo anchorage-independent growth. In contrast, c-Myc was sufficient to increase mammosphere formation by ∼2.3-fold. In addition, co-expression of c-Myc and H-Ras (G12V) maximally elevated mammosphere formation by ∼3.15-fold.

**Figure 3:**
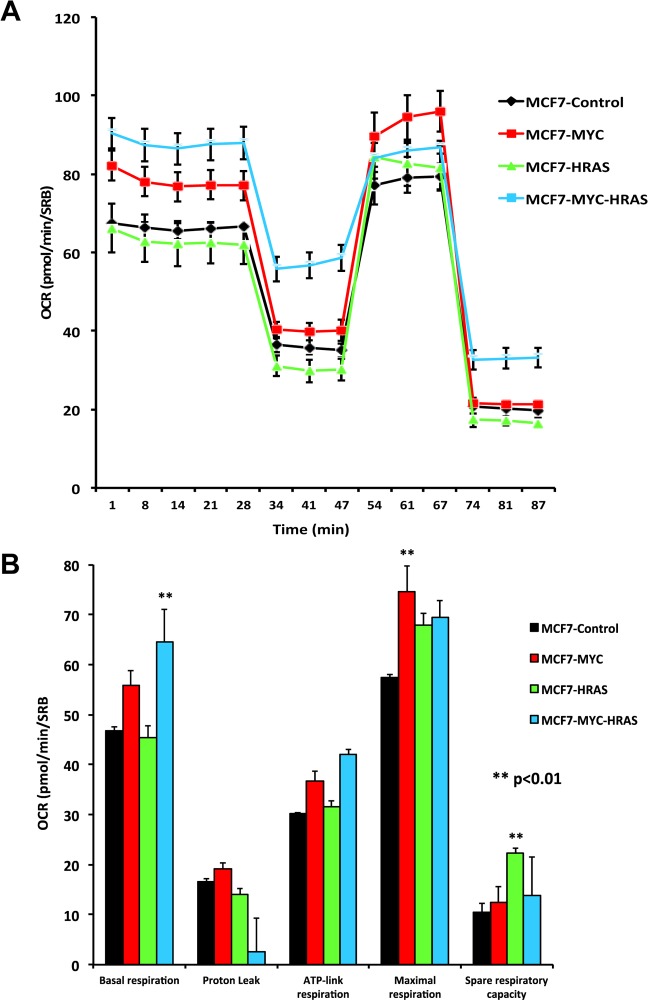
MYC-RAS co-operativity increases mitochondrial respiration Oxygen consumption rate (OCR) was measured in MCF7 cell lines, using the Seahorse XFe96 metabolic flux analyzer. Both (**A**) OCR metabolic tracings and (**B**) Bar graphs are shown. Note that c-Myc and c-Myc plus H-Ras (G12V) increase mitochondrial respiration rates.

**Figure 4:**
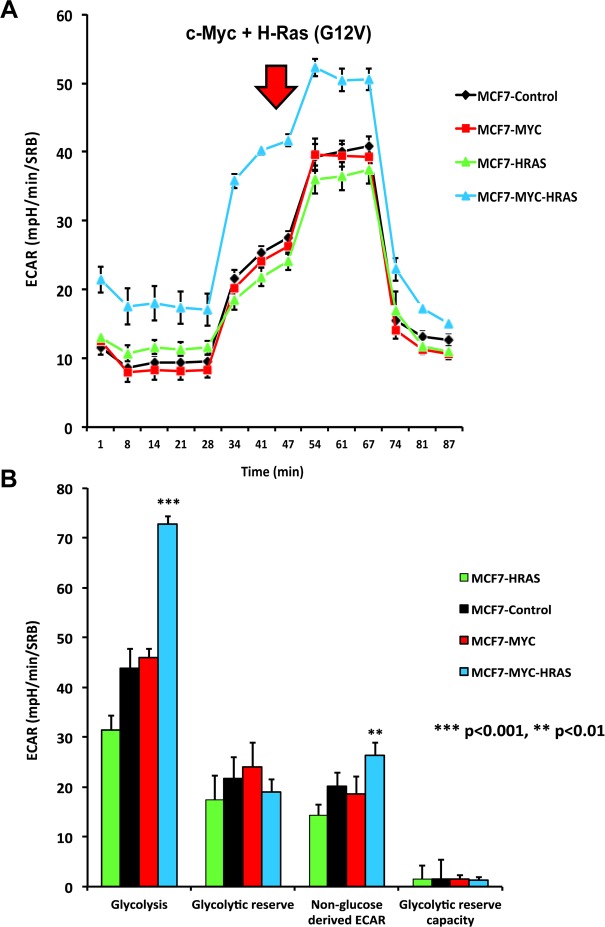
MYC-RAS co-operativity enhances glycolysis Extracellular acidification rate (ECAR) was measured in MCF7 cell lines, using the Seahorse XFe96 metabolic flux analyzer. Both (**A**) ECAR metabolic tracings and (**B**) Bar graphs are shown. Note that only transduction with c-Myc plus H-Ras (G12V) increases glycolytic rates.

To better understand how MYC-RAS co-operativity fuels CSC propagation, we next subjected the panel of MCF7 cells to metabolic phenotyping, using the Seahorse XFe96 metabolic flux analyzer. Figure 3 illustrates that c-Myc increases mitochondrial respiration, either alone or in combination with H-Ras (G12V). Interestingly, H-Ras (G12V) specifically increased glycolysis, but only in the cells transduced with c-Myc (Figure 4).

### Proteomics analysis identifies c-Myc targets: treatment with Doxycycline

Based on our initial findings, it appeared that c-Myc had the largest effects on both “stemness” and cellular metabolism, relative to H-Ras (G12V). Thus, we focused our efforts on c-Myc and performed an unbiased detailed proteomics analysis on MCF7 cells harboring c-Myc alone. These results are summarized in Table 1.

**Table 1:** Proteomics analysis of MCF7 cells transduced with c-Myc: focus on mitochondrial-related targets

Symbol	Gene Description	Fold-Upregulation
CHCHD2	Putative coiled-coil-helix-coiled-coil-helix domain-containing protein, mitochondrial	4.05
PRDX3	Thioredoxin-dependent peroxide reductase, mitochondrial	3.96
COX5A	Cytochrome c oxidase subunit 5A, mitochondrial	3.65
IMMT	Mitochondrial inner membrane protein	3.54
TRNT1	CCA tRNA nucleotidyltransferase 1, mitochondrial	3.22
TIMM44	Mitochondrial import inner membrane translocase subunit TIM44	3.15
C21orf33	ES1 protein homolog, mitochondrial (HES1)	3.00
AKAP1	A kinase anchor protein 1, mitochondrial	2.72
MDH2	Malate dehydrogenase, mitochondrial (EC 1.1.1.37)	2.69
IDH3A	Isocitrate dehydrogenase [NAD] subunit alpha, mitochondrial	2.64
TRAP1	Heat shock protein 75 kDa, mitochondrial	2.40
FASN	Fatty acid synthase	2.16
HADHB	Trifunctional enzyme subunit beta, mitochondrial	2.13
CLUH	Clustered mitochondria protein homolog/KIAA0664	2.12
ATP5I	ATP synthase subunit e, mitochondrial	1.98
ABAT	4-aminobutyrate aminotransferase, mitochondrial	1.97
CLPX	ATP-dependent Clp protease ATP-binding subunit clpX-like, mitochondrial	1.94
HSPD1	60 kDa heat shock protein, mitochondrial	1.89
SLIRP	SRA stem-loop-interacting RNA-binding protein, mitochondrial	1.81
TUFM	Elongation factor Tu, mitochondrial	1.75
COX5B	Cytochrome c oxidase subunit 5B, mitochondrial	1.69
PDHB	Pyruvate dehydrogenase E1 component subunit beta, mitochondrial	1.64
UQCR11	Cytochrome b-c1 complex subunit	1.64
SUCLG2	Succinyl-CoA ligase [GDP-forming] subunit beta, mitochondrial	1.60
NDUFS1	Mitochondrial NADH-ubiquinone oxidoreductase 75 kDa subunit	1.51
TOMM7	Mitochondrial import receptor subunit TOM7 homolog	1.49

Most notably, ∼26 mitochondrial proteins were elevated in MCF7-c-Myc cells, relative to vector alone control MCF7 cells. Importantly, many of these proteins are associated with either i) OXPHOS (COX5A, ATP5I, COX5B, UQCR11, and NDUFS1), ii) the TCA cycle (MDH2, IDH3A, PDHB, and SUCLG2), iii) mitochondrial biogenesis (IMMT, TIMM44, TRAP1, HSPD1, SLIRP, TUFM, and TOMM7), or iv) mitochondrial oxidative stress (CHCHD2 and PRDX3).

Therefore, we conclude that it may be possible to use mitochondrial inhibitors to target c-Myc amplification in CSCs. To test this hypothesis more directly, we used a well-known FDA-approved antibiotic (Doxycycline) that inhibits mitochondrial biogenesis as an off-target effect. Doxycycline is relatively non-toxic and has an excellent safety profile and is well-tolerated by patients, even during chronic usage.

**Figure 5:**
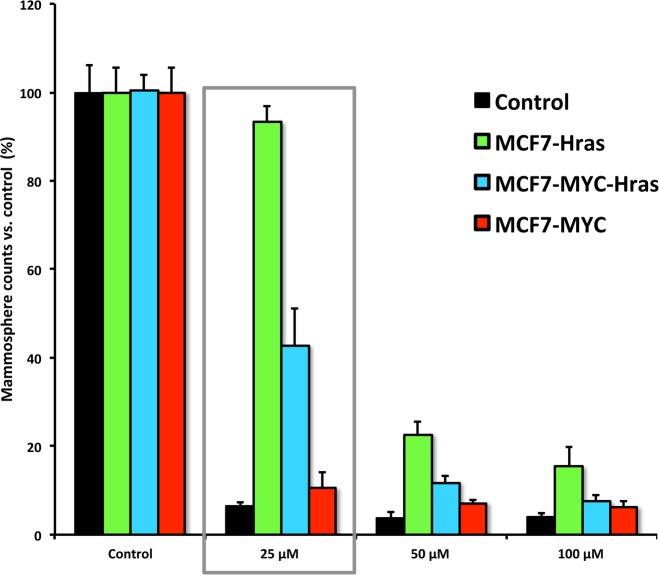
MYC-RAS-induced mammosphere formation remains sensitive to Doxycycline treatment Note that mammosphere formation in MCF7-H-Ras (G12V) cells is more resistant to Doxycycline, but this can be overcome by using slightly higher dosages.

As predicted, Figure 5 shows that MYC-RAS-induced mammosphere formation is sensitive to Doxycycline treatment. However, mammosphere formation in MCF7-H-Ras (G12V) cells is more resistant to Doxycycline, but this was easily overcome by using higher doses of Doxycycline. These findings provide further evidence that mitochondrial biogenesis is clearly important for promoting CSC propagation.

### Low-dose Rotenone augments mammosphere formation: Reversal with a mitochondrial anti-oxidant (Mito-tempo) or Doxycycline

Based on the proteomics analysis presented in Table 1, we observed that c-Myc induced the expression of a powerful mitochondrial anti-oxidant (PRDX3), as well as a key component of mitochondrial complex I (NDUFS1). Other components of complex III, IV and V were also up-regulated.

Importantly, PRDX3 is normally over-expressed in response to mitochondrial oxidative stress [6]. NDUFS1 is the core subunit of complex I; this protein product harbors both the NADH dehydrogenase and oxidoreductase activities [7]. NDUFS1 is the largest component of complex I and contains the iron-sulfur binding site [7].

Similarly, CHCHD2 expression was also strongly induced by c-Myc. Importantly, CHCHD2 has been suggested to function as a ROS scavenger, while at the same time optimizing OXPHOS and other anti-apoptotic mitochondrial functions [8].

Taken together, these findings are consistent with the idea that c-Myc induces “stemness” in part by driving and effectively managing the onset of mitochondrial oxidative stress.

To test this working hypothesis, we next used a pharmacological approach, to chemically stimulate mitochondrial oxidative stress. Rotenone is a naturally occurring isoflavone that behaves as an inhibitor of complex I, which strongly induces mitochondrial oxidative stress [9]. Rotenone is also an environmental pollutant, as it is frequently used as a broad-spectrum pesticide and insecticide [10]. During Rotenone treat- ment, mitochondrial complexes I and III are the major sites of electron leakage, resulting in increased superoxide production [11].

**Figure 6:**
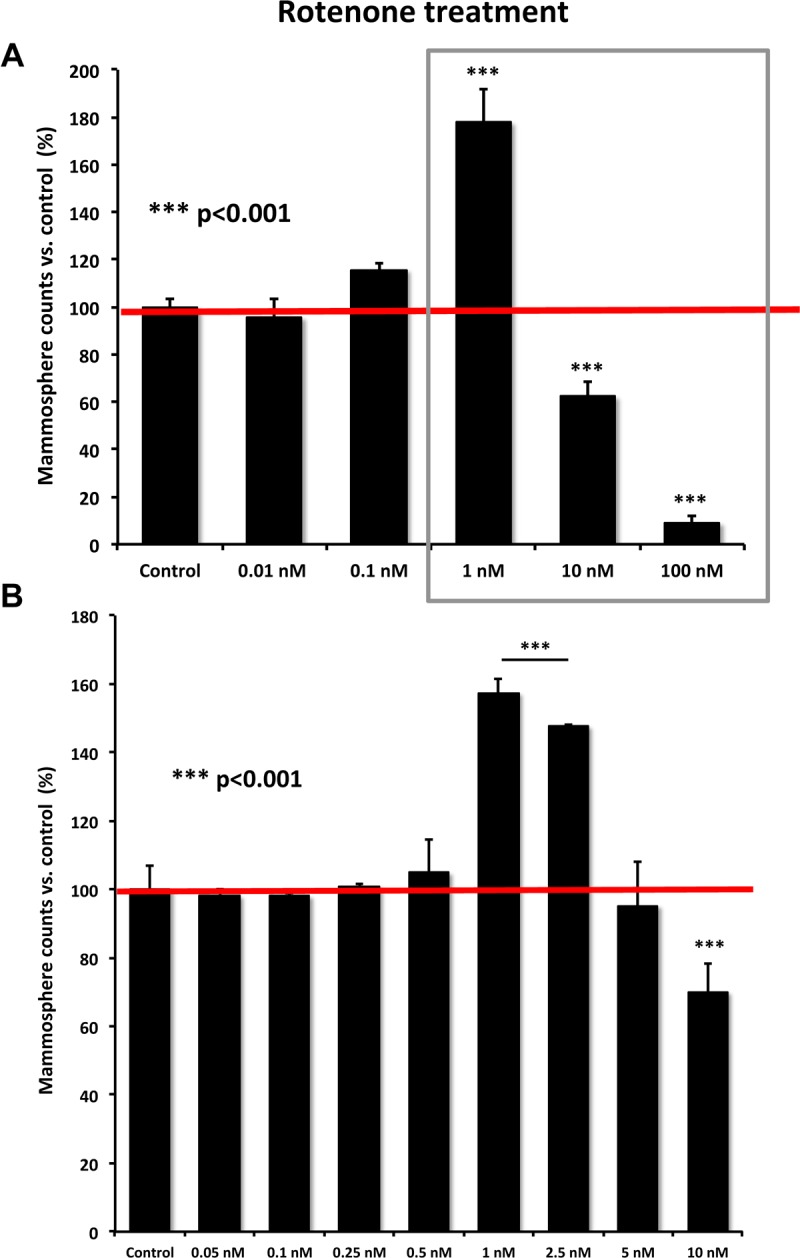
Rotenone dose-dependently stimulates mammosphere formation, in the low nanomolar range (**A**) Concentration range from 0.01 to 100 nM; (**B**) Concentration range from 0.05 to 5 nM. Note that low-dose Rotenone (1 nM and 2.5 nM) stimulates mammosphere formation, while higher concentrations of Rotenone (10-100 nM) are inhibitory.

We treated MCF7 cells with increasing concentrations of Rotenone. Interestingly, low-dose Rotenone (1 or 2.5 nM) was indeed sufficient to stimulate mammosphere formation, by ∼1.6 to 1.8-fold (Figure 6). However, higher doses of Rotenone (10-100 nM) inhibited mammosphere formation, as expected, due to lethal/toxic levels of oxidative stress.

**Figure 7:**
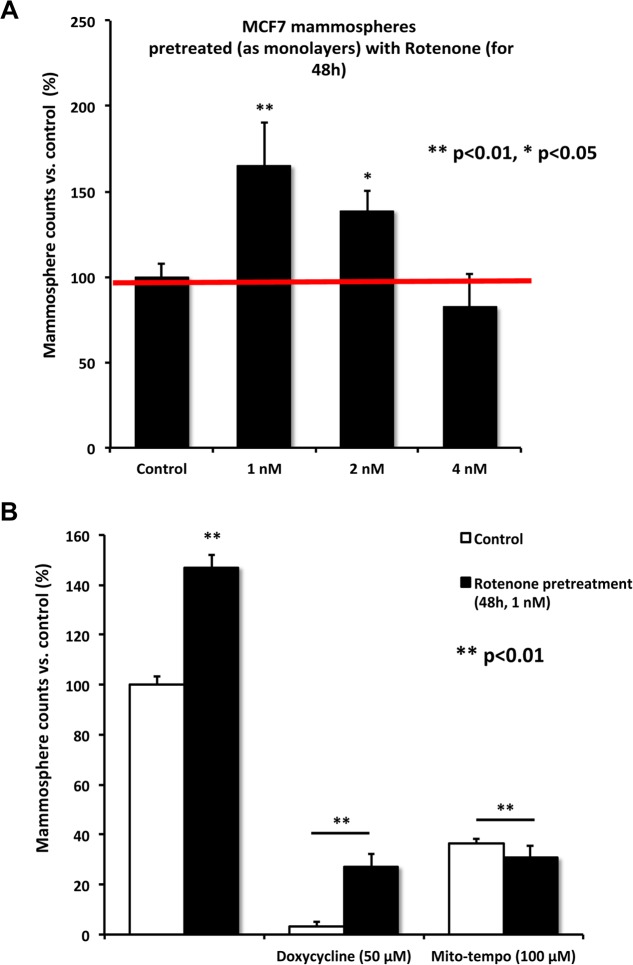
Rotenone-enhanced mammosphere formation is ablated by treatment with Doxycycline or Mito-tempo (**A**) MCF7 cells were pre-treated for 48 hours with Rotenone (from 1 to 4 nM) as a monolayer. Then the cells were harvested by trypsinization and re-plated under low-attachment conditions for the mammosphere assay. Note that pre-treatment of MCF7 monolayers with rotenone at either 1 or 2 nM stimulated mammosphere formation. (**B**) As in panel (**A**), except the MCF7 monolayers were pre-treated for 48 hours with Rotenone (1 nM). Then, the cells were re-plated under low-attachment conditions. Note that further treatment with i) an inhibitor of mitochondrial biogenesis (Doxycycline; 50 μM) or ii) a mitochondrial-based antioxidant (Mito-tempo; 100 μM), is sufficient to block Rotenone-enhanced mammosphere formation.

To further validate that the effects of low-dose Rotenone were due to oxidative stress, we also used a mitochon-drial-specific anti-oxidant, namely Mito-tempo. More specifically, Mito-tempo functions as a ROS scavenger, by targeting superoxide anions generated in mitochondria [12]. Figure 7 highlights that treatment with Mito-tempo (100 μM) was indeed sufficient to inhibit the stimulatory effects of Rotenone (1 nM) on MCF7 mammosphere formation. Virtually identical effects were also observed with Doxycycline (50 μM), which functions as a known inhibitor of mitochondrial biogenesis.

### Clinical relevance of c-Myc mitochondrial targets in human breast cancers

To determine the potential clinical relevance of our findings, we next assessed whether the c-Myc mitochondrial targets that we identified in MCF7 cells were also transcriptionally up-regulated in human breast cancer cells *in vivo*.

For this purpose, we employed a published clinical data set of N=28 breast cancer patients in which their tumor samples were subjected to laser-capture micro-dissec- tion, to physically separate epithelial cancer cells from their adjacent tumor stroma. Table 2 presents a summary of these findings.

**Table 2:** Myc-targets transcriptionally up-regulated in human breast cancer in vivo (Cancer Epithelia vs. Tumor Stroma)

Symbol	Gene Description	Fold-Upregulation	P-value
CHCHD2	Putative coiled-coil-helix domain-containing protein, mitochondrial	5.79	1.85E-07
COX5B	Cytochrome c oxidase subunit 5B, mitochondrial	5.03	2.86E-06
PRDX3	Thioredoxin-dependent peroxide reductase, mitochondrial	4.99	3.30E-06
IMMT	Mitochondrial inner membrane protein	4.71	8.89E-06
PDHB	Pyruvate dehydrogenase E1 component subunit beta, mitochondrial	4.51	1.75E-05
MDH2	Malate dehydrogenase, mitochondrial (EC 1.1.1.37)	4.18	5.32E-05
COX5A	Cytochrome c oxidase subunit 5A, mitochondrial	3.62	3.22E-04
C21orf33	ES1 protein homolog, mitochondrial (HES1)	3.60	3.49E-04
UQCR11	Cytochrome b-c1 complex subunit (UQCR)	3.43	5.87E-04
HSPD1	60 kDa heat shock protein, mitochondrial	3.42	5.93E-04
TUFM	Elongation factor Tu, mitochondrial	3.38	6.74E-04
AKAP1	A kinase anchor protein 1, mitochondrial	3.33	7.75E-04
NDUFS1	Mitochondrial NADH-ubiquinone oxidoreductase 75 kDa subunit	3.20	1.15E-03
HADHB	Trifunctional enzyme subunit beta, mitochondrial	3.06	1.73E-03
SUCLG2	Succinyl-CoA ligase [GDP-forming] subunit beta, mitochondrial	3.03	1.89E-03
TOMM7	Mitochondrial import receptor subunit TOM7 homolog	3.03	1.85E-03
ATP5I	ATP synthase subunit e, mitochondrial	3.01	1.97E-03
IDH3A	Isocitrate dehydrogenase [NAD] subunit alpha, mitochondrial	2.16	1.78E-02
CLPX	ATP-dependent Clp protease ATP-binding subunit clpX-like, mitochondrial	2.11	1.96E-02
ABAT	4-aminobutyrate aminotransferase, mitochondrial	2.08	2.14E-02

Transcriptional profiling data derived from the analysis of N=28 breast cancer patients are shown, high-lighting the levels of fold-upregulation observed in the epithelial cancer cell compartment (relative to the tumor stroma), and corresponding p-values derived from the analysis of these clinical samples.

Overall, nearly 80% of the c-Myc mitochondrial targets that we identified in MCF7 cells *in vitro* were also transcriptionally elevated in human breast cancer cells *in vivo* (20 out of 26, ∼77%). This observation provides a strong indication that the mitochondrial Myc-targets that we identified here are of high trans-lational and clinical relevance, for improving patient benefit.

### Prognostic value of c-Myc-induced mitochondrial targets in human breast cancer patients: Implications for treatment failure and distant metastasis

**Figure 8:**
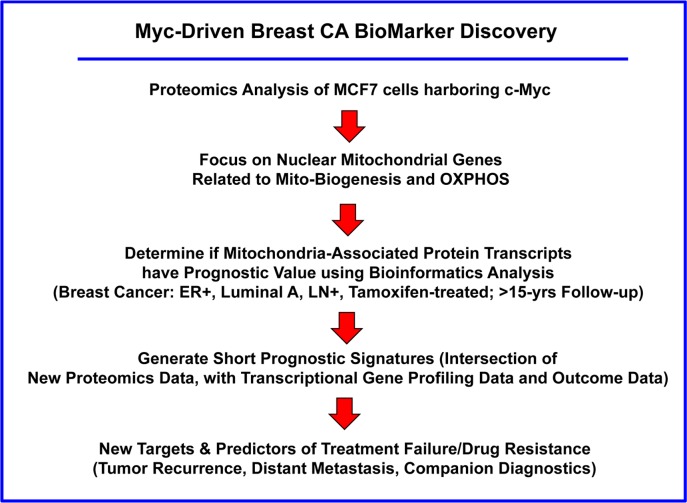
Flow-diagram illustrating our overall approach to Myc-driven biomarker discovery In this analysis, we focused specifically on ER(+) patients, luminal A sub-type, that were lymph-node positive (LN(+)) at diagnosis, who were treated with tamoxifen and followed over a period of nearly 200 months (>15 years). In this context, we evaluated the prognostic value of Myc-related mitochondrial markers for predicting tumor recurrence (RFS) or distant metastasis (DMFS), in this patient population.

Next, we assessed the prognostic significance of the c-Myc-induced mitochondrial targets that we identified by proteomics analysis. A work-flow diagram briefly illustrating our overall informatics-based approach to oncogene-driven breast cancer biomarker discovery is shown in Figure 8.

More specifically, we used publically available transcriptional profiling data from the tumors of breast cancer patients that were treated with tamoxifen, but did not receive any chemotherapy. For this purpose, we selected high-risk patients that were lymph-node positive at diagnosis, and we focused on the luminal A subtype, which represents the most common form of ER(+) breast cancers (N=152 patients). Using this systematic approach, we identified 5 gene transcripts and 9 gene probes that showed high prognostic value (Table 3).

**Table 3:** Prognostic value of individual c-Myc targets in human breast cancer patients

Symbol	Gene Probe	HR (Hazard Ratio)	P-value (Log Rank Test)
HSPD1	200807_s_at	3.46	1.3e-05
HSPD1	200806_s_at	2.34	0.0049
TIMM44	203093_s_at	2.51	0.0042
COX5B	213735_s_at	2.51	0.0012
COX5B	202343_x_at	2.30	0.0032
COX5B	211025_x_at	2.13	0.0077
IDH3A	202069_s_at	2.46	0.0026
IDH3A	202070_s_at	2.25	0.0089
TRAP1	221235_s_at	1.77	0.048

In order to increase the prognostic power of these individual mitochondrial biomarkers, we next selected the most promising ones and used them to create a new c-Myc-based Mito-Signature that contains 3 genes (Summarized in Tables 4 & 5). This Mito-Signature effectively combines: i) a mitochondrial chaperone (HSPD1), with ii) an OXPHOS subunit (COX5B; from Complex IV) and iii) a marker of mitochondrial biogenesis (TIMM44; Translocase Of Inner Mitochondrial Membrane 44).

**Table 4:** Tumor recurrence: prognostic value of a c-Myc-based mito-signature in human breast cancer patients

Symbol	Gene Probe	HR (Hazard Ratio)	P-value (Log Rank Test)
HSPD1	200807_s_at	3.46	1.3e-05
COX5B	213735_s_at	2.51	0.0012
TIMM44	203093_s_at	2.51	0.0042
**Combined**		**4.69**	**2.4e-08**

ER(+), Luminal A, LN(+), treated with anti-estrogen (mostly Tamoxifen); RFS, Relapse-Free Survival; N=152

**Table 5:** Distant metastasis: prognostic value of a c-Myc-based mito-signature in human breast cancer patients

Symbol	Gene Probe	HR (Hazard Ratio)	P-value (Log Rank Test)
HSPD1	200807_s_at	3.50	9.7e-05
COX5B	213735_s_at	3.20	0.00075
TIMM44	203093_s_at	3.06	0.0024
**Combined**		**4.94**	**2.8e-07**

ER(+), Luminal A, LN(+), treated with anti-estrogen (mostly Tamoxifen); DMFS, Distant Metastasis-Free Survival; N=149

**Figure 9:**
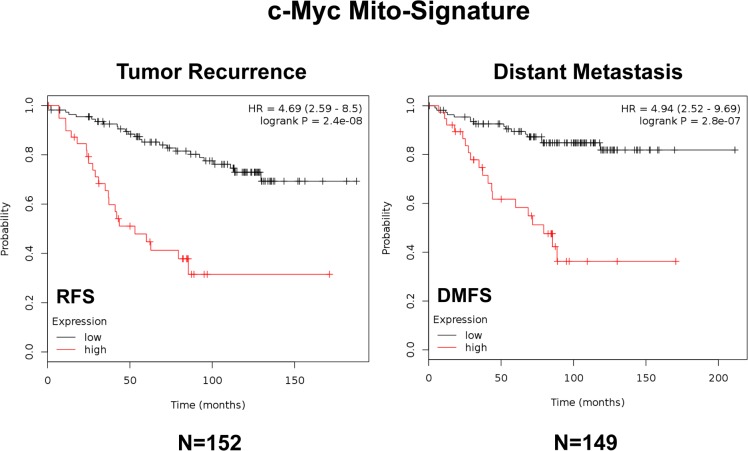
A short c-Myc-related mitochondrial signature predicts poor clinical outcome in high-risk ER(+) breast cancer patients Note that this short 3-gene signature (HSPD1/COX5B/TIMM44) effectively predicts tumor recurrence and distant metastasis in ER(+)/LN(+)/luminal A patients treated with tamoxifen therapy, indicative of treatment failure and tamoxifen-resistance. RFS, recurrence-free survival; DMFS, distant metastasis-free survival.

Briefly, Figure 9 (left panel) shows the results of this K-M analysis for relapse-free survival (RFS) for the Myc-based Mito-Signature (HR=4.69; p=2.4e-08). Similar results were also obtained for distant metastasis-free survival (DMFS) (HR=4.94; p=2.8e-07) (Figure 9, right panel). Therefore, this Myc-based Mito-Signature was very effective at predicting tamoxifen-resistance and treatment failure for endocrine therapy.

**Figure 10:**
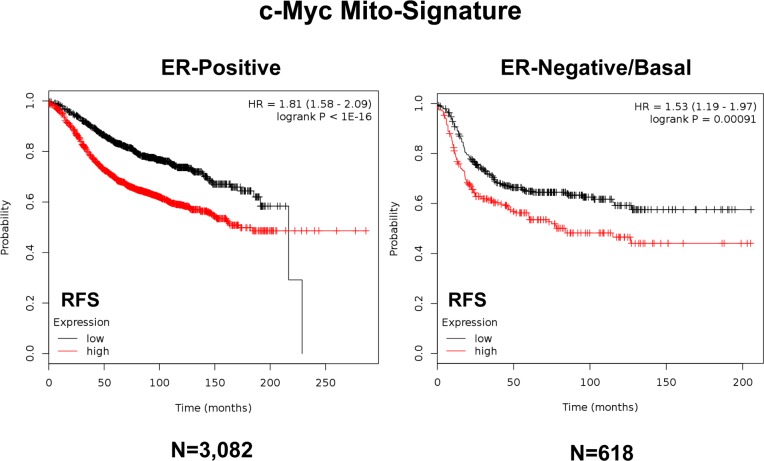
A short c-Myc-related mitochondrial signature predicts poor clinical outcome, in both ER(+) and ER(−) breast cancer patients Note that this short 3-gene signature (HSPD1/COX5B/TIMM44) also effectively predicts tumor recurrence in larger populations of both ER(+) [N=3,082] and ER(−) [N=618] breast cancer patients. RFS, recurrence-free survival.

In addition, we directly assessed the behavior of the Myc-based Mito-Signature in even larger and more varied patient populations, where the therapy was not restricted to tamoxifen. Importantly, Figure 10 illustrates that the Myc Mito-Signature was also effective in both ER(+) (N=3,082; HR=1.81; p<1e-16) and ER(−)/basal (N=618; HR=1.53; p=0.00091) breast cancer populations, as well as in all breast cancer subtypes combined (N=3,951; HR=1.71; p<1e-16; See Supplementary Figure S1).

As such, this Myc-based mitochondrial gene signature may represent an important new prognostic tool for predicting patient outcomes, in a wide variety of different breast cancer patient populations, as well as in ER(+) patients being treated with hormonal therapies.

## DISCUSSION

### MYC/RAS co-operativity: focus on stemness and cellular metabolism

To mechanistically determine exactly how MYC/RAS co-operativity contributes to stemness in breast cancer cells, we used MCF7 cells as a model system. Briefly, we generated a panel of MCF7 cell lines transduced with c-Myc or H-Ras (G12V), either individually or in combination. In this context, the mammosphere assay (3D spheroid formation) was used as a standard measure of CSC activity. c-Myc increased both mitochondrial respiration and mammosphere formation, without changing glycolytic flux. Surprisingly, H-Ras (G12V) alone did not affect glycolysis or either mammosphere formation. Interestingly, H-Ras (G12V) stimulated both glycolysis and mammosphere formation, but only when combined with c-Myc.

These findings provide novel insight into how MYC/RAS co-operativity synergistically drives changes in stemness and cellular metabolism. Since it is well-established that c-Myc exerts some of its effects by increasing mitochondrial biogenesis, we also determined the activity of another stimulus that drives mitochondrial biogenesis, namely oxidative stress.

### A phenotypic approach to cancer therapy: targeting mitochondrial biogenesis

To chemically induce mitochondrial oxidative stress, we used a well-known mitochondrial poison (Rotenone) to inhibit the function of mitochondrial complex I. Rotenone treatment yielded bi-phasic effects; low-dose Rotenone (1-2.5 nM) increased mammosphere formation, while higher doses (10-100 nM) were inhibitory. Next, we experimentally confirmed the hypothesis that mitochondrial biogenesis was absolutely necessary to stimulate CSC propagation, by employing Doxycycline, a safe FDA-approved inhibitor of mito-chondrial protein translation [13].

**Figure 11:**
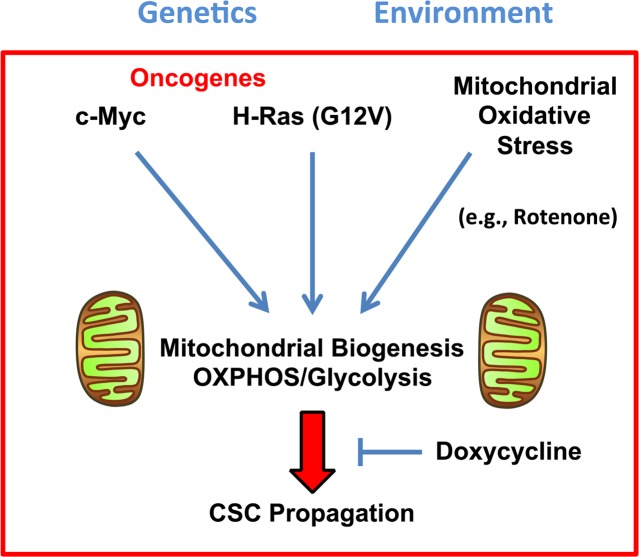
MYC-RAS co-operativity “fuels” stemness in cancer cells: Mutation-independent cancer therapy, with Doxycycline Here, we show that either a i) *genetic stimulus* [oncogene activation (c-Myc or H-Ras (G12V)] or an ii) *environmental stimulus* [mitochondrial oxidative stress (chemically induced by Rotenone)] are both sufficient to drive metabolic reprogramming and increased CSC propagation. Nevertheless, the positive growth effects of these oncogenic stimuli can both be blocked using a mutation-independent or “phenotypic approach”, by employing Doxycycline.

Doxycycline treatment was sufficient to block the positive effects of H-Ras (G12V), c-Myc, and Rotenone on CSC propagation (Figure 11). As such, our results with Doxycycline treatment provide an impetus for the creation of novel therapies to target mitochondrial protein translation in CSCs.

Our results also directly support the idea of a “mutation-independent” approach to cancer therapy. Importantly, most effective cancer therapies used today target “cancer cell behaviors”, such as increased cell proliferation, rather than particular genetic mutations. Thus, we propose that a cell-based “phenotypic approach” towards generating new cancer therapies would be a more fruitful endeavor.

Interestingly, previous studies conducted by Porporato et al., 2014, have reported that the pre-treatment of B16F10 melanoma cells with low-dose Rotenone (in the 10-100 nM range) for only 6 hours resulted in dramatic increases in cell migration, invasion and lung metastasis [14]. Importantly, this pro-metastatic effect of short exposure to low-dose Rotenone could be prevented or reversed by employing Mito-tempo, a mitochondrial-specific anti-oxidant targeting superoxide anions [15].

These results are consistent with our current findings, although these authors did not mechanistically determine the effects of Rotenone on CSC propagation. Also, these authors did not examine the effects of Doxycycline on the pro-metastatic activity conferred by Rotenone pre-treatment. However, taken together, these data suggest that Doxycycline treatment may be very useful for preventing the development of metastatic disease.

In direct support of this assertion, earlier studies have shown that Doxycycline treatment can effectively prevent bone metastasis in a preclinical animal model, using MDA-MB-231 human breast cancer cells [16]. Therefore, further experimentation and clinical trials may be warranted, to explore the role of Doxycycline in the prevention and treatment of metastatic disease.

### Biomarker discovery & companion diagnostics: a “Proteomics-to-Genomics (PTG)” approach for in silico validation

Here, we used unbiased proteomics analysis of MCF7 cells stably transduced with c-Myc to identify new mitochondrial targets in human breast cancer cells. Using this approach, we identified 26 mitochondrial proteins that were specifically up-regulated in MCF7-c-Myc cells, relative to MCF7 control cells transduced with the vector alone.

To validate the translational and clinical relevance of our findings, we next intersected this proteomics data set, with independent transcriptional profiling data obtained via the laser capture of human breast cancer cells, from excised tumor tissue. Interestingly, our results indicated that 20 out of 26 proteins that were up-regulated in MCF7-c-Myc cells were also transcript-tionally up-regulated in human breast cancer cells, relative to adjacent tumor stromal cells.

**Figure 12:**
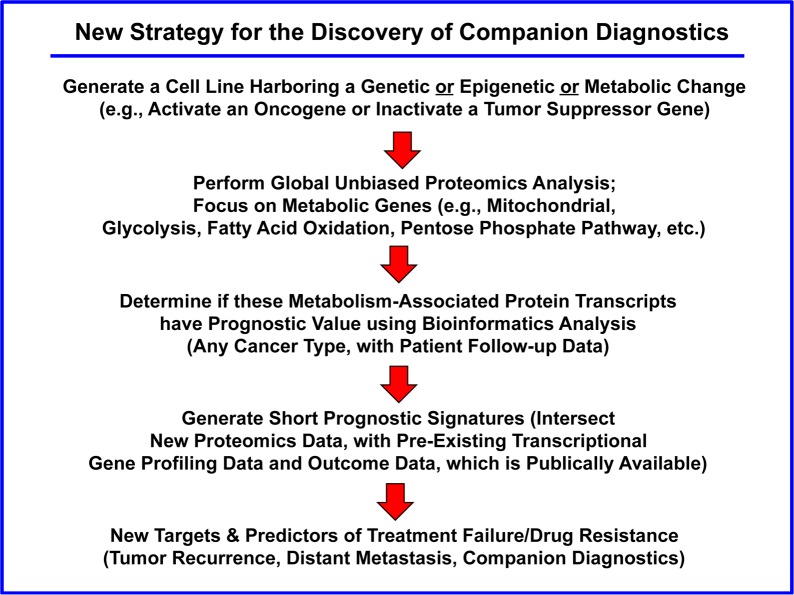
Diagram summarizing our new general strategy for the discovery of companion diagnostics This simplified strategy can be potentially applied to any cancer type. The first step involves the generation of a novel cellular model, which is interrogated by proteomics analysis. Then, these results are used to establish the prognostic value of these candidate biomarkers, by searching pre-existing human transcriptional profiling data, linked to clinical outcome (in silico validation). The prognostic value of these biomarkers can also be enhanced significantly, by using more than one marker in combination, forming a short signature. This “Proteomics-to-Genomics (PTG)” approach then efficiently yields new targets and biomarkers, linked to parameters associated with clinical outcome (tumor recurrence, distant metastasis, overall survival, or response to therapy).

**Figure 13:**
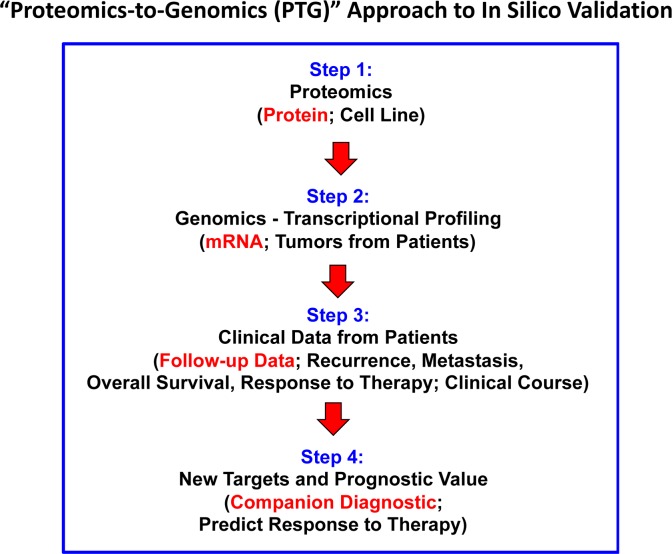
The “Proteomics‐to‐Genomics (PTG)” approach for the *in silico* validation of new biomarkers and novel drug targets In this flow‐diagram, proteomics data from a cellular model is directly used to interrogate existing genomics data (transcriptional profiling) from clinical samples, which are linked to follow‐up data. This approach directly allows for the *in silico* validation of i) the prognostic value (of a given candidate biomarker) and ii) helps to generate new targets for drug discovery, by directly demonstrating their clinical relevance.

To provide *in silico* validation of the prognostic value of these Myc-based targets, we also intersected our proteomic data with transcriptional profiling data obtained from tumor samples, linked to clinical outcome (Figures 12 and 13). This “Proteomics-to-Genomics (PTG)” approach allowed us to determine the prognostic value of these 26 mitochondrial proteins, either individually and/or in various combinations. Using this systematic approach, we identified 5 gene transcripts and 9 gene probes that showed high prognostic value. The prognostic value of these bio-markers was also enhanced significantly, by using more than one mitochondrial marker in combination, by forming a short signature.

Remarkably, this Myc-based Mito-Signature was very effective at predicting tamoxifen-resistance and treatment failure for endocrine therapy in ER(+) breast cancer patients. Importantly, this Myc-based Mito-Signature was also effective in larger cohorts of both ER(+) (N=3,082) and ER(−) (N=618) breast cancer patients. As a result, this Myc-based mitochondrial gene signature may represent an important new companion diagnostic for predicting patient outcomes and the response to anti-mitochondrial therapy, in a wide variety of different breast cancer patient populations.

### Advantages to using the “Proteomics-to-Genomics (PTG)” strategy

**Figure 14:**
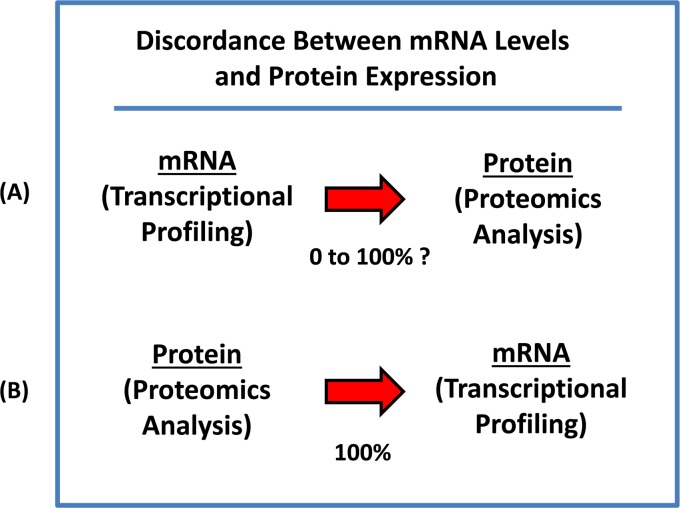
mRNA levels do not correlate with protein levels, creating a bottle-neck for protein biomarker discovery (**A**) Concordance between mRNA and protein is actually quite variable and completely unpredictable, ranging anywhere between 0 and 100%. This discordance between mRNA and protein expression levels ultimately makes it very difficult or nearly impossible, to use transcriptional profiling data for the development of new protein biomarkers as companion diagnostics. (**B**) Our new “Proteomics-to-Genomics (PTG)” strategy provides a simple straightforward solution to this practical problem. By starting out with proteomics data first and then integrating it with existing transcriptional profiling data, this allows one to quickly identify and select a sub-set of genes, with tight correlations, nearing 100%. It essentially allows one to “work-backwards”, providing a much needed systematic “short-cut” to protein biomarker discovery.

The “central dogma of molecular biology” teaches us that i) genes are encoded within genomic DNA, ii) that is first transcribed into messenger RNA (mRNA) by RNA-polymerase, iii) which is then later translated into protein by ribosomes (Figure 14). Thus, one would initially assume or predict that mRNA levels would directly correlate with protein levels. However, if you experimentally construct a plot of the levels of an mRNA species encoding a given gene product, versus its actual protein levels, the correlation coefficient (R) is normally less than 0.50 or 50% [17-21]. As such, the concordance between mRNA and protein is actually quite variable and completely unpredictable, ranging anywhere between 0 and 100% [17-21]. This discordance between mRNA and protein expression levels ultimately makes it very difficult or nearly impossible, to use transcriptional profiling data for the development of new protein biomarkers as companion diagnostics.

Our new “Proteomics-to-Genomics (PTG)” strategy provides a simple straightforward solution to this practical problem. By starting out with proteomics data first and then integrating it with existing transcriptional profiling data, this allows one to quickly identify and select a sub-set of genes, with tight correlations, nearing 100%.

With this approach, one already knows *a priori* that the protein product is over-expressed, even before correlating it with transcriptional profiling data. It essentially allows one to “work-backwards”, providing a much needed systematic “short-cut” to protein biomarker discovery. This could help remove the bottle-neck on the route to successful companion diagnostics.

### Implications for understanding oncogenic stress and senescence

Oncogene expression in normal cells results in excessive cellular stress, usually leading to ROS over-production, driving either i) cell death via apoptosis, ii) cell-cycle arrest via senescence or iii) the activation of autophagic/mitophagic survival programs [22-24]. One important mechanism by which CSCs escape from oncogenic stress is through cell immortalization, via the over-expression or activation of telomerase activity (e.g., hTERT) [22-24]. This may ultimately explain why stem cells are the ultimate target of oncogenes, as non-stem cells cannot survive oncogenic stress, because they lack sufficient telomerase activity. Oncogenes simply kill most non-stem cells, leaving behind only surviving stem-like cells, which are the only cell type that is resistant to, or effectively tolerates, oncogenic stress.

Recently, we showed that CSCs with high telomerase activity also show high mitochondrial mass. Interest-ingly, the survival effects conferred by hTERT-expression in CSCs could be overcome, simply by treatment with an inhibitor of mitochondrial biogenesis, namely Doxycycline [25]. Similar results were obtained with Palbociclib, a CDK4/6 inhibitor [25]. Therefore, the survival effects conferred by telomerase in CSCs can be mechanistically reversed by chemically inducing cell-cycle arrest (Palboiclib) or mitochondrial dys-function (Doxycycline).

In summary, inhibition of either “cellular proliferation” or “mitochondrial proliferation” is sufficient to block CSC propagation. These observations provide further experimental support for a mutation-independent approach to cancer therapy, by phenotypically targeting CSCs.

## MATERIALS AND METHODS

### Materials

MCF7 cells were purchased from the ATCC (American Type Culture Collection). Gibco-brand cell culture media (DMEM) was purchased from Life Technologies.

### Lentiviral gene transduction

Lentiviral plasmids, packaging cells and reagents were purchased from Genecopoeia, Inc. Forty-eight hours after seeding, 293Ta packaging cells were transfected with lentiviral vectors encoding c-Myc, H-Ras (G12V) or the empty vector (EX-NEG-Lv105-PURO), using Lenti-PacTM HIV Expression Packaging Kit, according to the manufacturer's instructions. Two days post-transfection, lentivirus-containing culture medium was passed through a 0.45 μm filter and added to the target cells (MCF-7 cells) in the presence of 5 μg/ml Polybrene. Infected cells were selected with a con-centration of 1.5 μg/ml of puromycin. After puromycin-resistant cells were growing continuously, a population of MYC transfected cells or control cells were further infected with medium of 293Ta cells transfected with an H-Ras (G12V) lentiviral vector or empty vector (EX-NEG-Lv151-NEO) (virus produced, as same above). Double-transfected c-Myc/H-Ras (G12V) MCF7 cells were then selected with Geneticin (G418). These MCF7 cell lines were originally generated at the University of Manchester.

### Mammosphere formation assay

A single cell suspension of MCF7 cells was prepared using enzymatic (1x Trypsin-EDTA, Sigma Aldrich) and manual disaggregation with a 25 gauge needle. Cells were then plated at a density of 500 cells/cm^2^ in mammosphere medium (DMEM-F12/ B27 / 20-ng/ml EGF/PenStrep) in non-adherent conditions, in culture dishes coated with (2-hydroxyethylmethacrylate) (poly-HEMA, Sigma). Cells were grown for 5 days and maintained in a humidified incubator at 37°C at an atmospheric pressure in 5% (v/v) carbon dioxide/air. After 5 days in culture, spheres >50 μm were counted using an eye-piece graticule, and the percentage of cells plated which formed spheres was calculated and is referred to as percent mammosphere formation, nor-malized to vehicle-alone treated controls. Mammo-sphere assays were performed in triplicate and repeated three times independently.

### Seahorse XFe96 metabolic flux analysis

Extracellular acidification rates (ECAR) and real-time oxygen consumption rates (OCR) for MCF7 cells were determined using the Seahorse Extracellular Flux (XF96) analyzer (Seahorse Bioscience, MA, USA). MCF7 cells were maintained in DMEM supplemented with 10% FBS (fetal bovine serum), 2 mM GlutaMAX, and 1% Pen- Strep. 8,000 cells per well were seeded into XF96-well cell culture plates, and incubated overnight at 37°C in a 5% CO2 humidified atmosphere. Next day, cells were washed in pre-warmed XF assay media (for OCR measurement, XF assay media was supplemented with 10mM glucose, 1mM Pyruvate and adjusted at pH 7.4). Cells were then maintained in 175 μL/well of XF assay media at 37°C, in a non-CO2 incubator for 1h. During incubation, 25 μL of of 80mM glucose, 9μM oligomycin, 1M 2-deoxyglucose (for ECAR measurement) and 25 μL of 10μM oligomycin, 9μM FCCP, 10μM rotenone, 10μM antimycin A (for OCR measurement) in XF assay media was loaded into the injection ports of the XFe-96 sensor cartridge. During the experiment, the instrument injected these inhibitors into the wells at a given time point, while ECAR/OCR was measured continuously. ECAR and OCR measurements were normalized by protein content (Sulphorhodamine B assay). Data sets were analyzed by XFe-96 software, using one-way ANOVA and Student's t-test calculations. All experiments were performed in triplicate.

### Global semi-quantitative proteomics analysis

Cell lysates were prepared for trypsin digestion by sequential reduction of disulphide bonds with TCEP and alkylation with MMTS. Then, the peptides were extracted and prepared for LC-MS/MS. All LC-MS/MS analyses were performed on an LTQ Orbitrap XL mass spectrometer (Thermo Scientific, San Jose, CA) coupled to an Ultimate 3000 RSLCnano system (Thermo Scientific, formerly Dionex), The Netherlands). Xcalibur raw data files acquired on the LTQ-Orbitrap XL were directly imported into Progenesis LCMS software (Waters Corp., Milford, MA, formerly Nonlinear dynamics, Newcastle upon Tyne, UK) for peak detection and alignment. Data were analyzed using the Mascot search engine. Five technical replicates were analyzed for each MCF7 sample (c-Myc transduced vs. Empty vector control cells).

### Data mining

To firmly establish the clinical relevance of our results from the proteomics analysis of MCF7 cells harboring c-Myc, we re-analyzed the transcriptional profiles of epithelial breast cancer cells and adjacent tumor stromal cells that were physically separated by laser-capture microdissection (from N=28 human breast cancer patients) [26].

### Kaplan-Meier (K-M) analyses

To perform K-M analysis nuclear mitochondrial gene transcripts, we used an open-access online survival analysis tool to interrogate publically available microarray data from up to 3,951 breast cancer patients [27]. This allowed us to determine their prognostic value. For this purpose, we primarily analyzed data from ER(+) patients that were LN(+) at diagnosis and were of the luminal A sub-type, that were primarily treated with tamoxifen and not other chemotherapy (N=152 patients). In this group, 100% the patients received some form of hormonal therapy and >90% of them received tamoxifen. Biased and outlier array data were excluded from the analysis. Hazard-ratios were calculated, at the best auto-selected cut-off, and p-values were calculated using the logrank test and plotted in R. K-M curves were also generated online using the K-M-plotter (as high-resolution TIFF files), using univariate analysis: http://kmplot.com/analysis/index.php?p=service&cancer=breast

This allowed us to directly perform *in silico* validation of these mitochondrial biomarker candidates. The multi-gene classifier function of the program was used to test the prognostic value of short mitochondrial gene signatures, using the mean expression of the selected probes. The most updated version of the database was utilized for these analyses; however, these studies were originally performed in 2014 and virtually identical results were obtained with the 2012 and 2014 versions of the database.

### Statistical analyses

Statistical significance was determined using the Student's t-test, values of less than 0.05 were considered significant. Data are shown as the mean ± SEM, unless stated otherwise.

## SUPPLEMENTARY MATERIAL FIGURE


