# Supplementary material for: A new mutation-independent approach to cancer therapy: Inhibiting oncogenic RAS and MYC, by targeting mitochondrial biogenesis
Source: Aging (Albany NY). 2017 Oct 27;9(10):2098–116. doi: 10.18632/aging.101304 (PMC5680558; doi:10.18632/aging.101304)
Supplement: Supplementary file 1 [file aging-09-2098-s001.pdf]

c-Myc Mito-Signature

All Breast Cancer Subtypes Combined

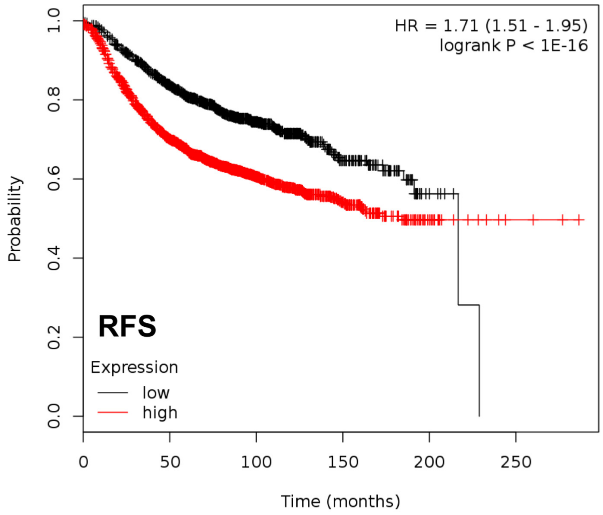

Supplementary Figure S1. A short c-Myc-related mitochondrial signature predicts poor clinical outcome, in all breast cancer sub-types, when combined. Note that this short 3-gene signature (HSPD1/COX5B/TIMM44) also effectively predicts tumor recurrence in a larger population of breast cancer patients (N=3,951). RFS, recurrence-free survival.
